# Imaging for Plasma Cell Dyscrasias: What, When, and How?

**DOI:** 10.3389/fonc.2022.825394

**Published:** 2022-03-24

**Authors:** Amrita Guha, Antariksh Vijan, Ujjwal Agarwal, Jayant Sastri Goda, Abhishek Mahajan, Nitin Shetty, Navin Khattry

**Affiliations:** ^1^ Department of Radiodiagnosis and Imaging, Tata Memorial Hospital, Mumbai, India; ^2^ Homi Bhabha National Institute, Training School Complex, Mumbai, India; ^3^ Department of Radiation Oncology, Advanced Centre for Treatment, Research and Education in Cancer, Tata Memorial Centre, Homi Bhabha National Institute, Mumbai, India; ^4^ Department of Medical Oncology, Advanced Centre for Treatment, Research and Education in Cancer, Tata Memorial Centre, Mumbai, India

**Keywords:** multiple myeloma, PET/CT, POEMS syndrome, computed tomography, low-dose CT, whole-body MRI

## Abstract

Imaging plays a vital role in the diagnosis, response assessment, and follow-up of patients with plasma cell bone disease. The radiologic diagnostic paradigm has thus far evolved with developing technology and availability of better imaging platforms; however, the skewed availability of these imaging modalities in developed vis-à-vis the developing countries along with the lack of uniformity in reporting has led to a consensus on the imaging criteria for diagnosing and response assessment in plasma cell dyscrasia. Therefore, it is imperative for not only the radiologists but also the treating oncologist to be aware of the criteria and appropriate imaging modality to be used in accordance with the clinical question. The review will allow the treating oncologist to answer the following questions on the diagnostic, prognostic, and predictive abilities of various imaging modalities for plasma cell dyscrasia: a) What lesions can look like multiple myeloma (MM) but are not?; b) Does the patient have MM? To diagnose MM in a high-risk SMM patient with clinical suspicion, which modality should be used and *why*?; c) Is the patient responding to therapy on follow-up imaging once treatment is initiated?; d) To interpret commonly seen complications post-therapy, when is it a disease and when is the expected sequel to treatment? Fractures, red marrow reconversion?; and e) When is the appropriate time to flag a patient for further workup when interpreting MRI spine done for back pain in the elderly? How do we differentiate between commonly seen osteoporosis-related degenerative spine versus marrow infiltrative disorder?

## Introduction

Multiple myeloma is a plasma cell dyscrasia characterized by abnormal monoclonal proliferation of plasma cells, approximately accounting for 10% of all hematological malignancies (1, 2). Although a disease of the elderly, it sometimes afflicts young adults (3). The disease is understood to progress in a continuum (diagnostic criteria listed **Table 1**); at one end of the spectrum are MGUS (monoclonal gammopathy of undetermined significance)-like benign conditions having a low risk of progression to multiple myeloma (MM), to the premalignant SMM (smoldering MM) (4) with an approximately 10% risk of progression, to the frank multiple myeloma with end-organ damage at the other end of the spectrum (5). The “CRAB” features as listed in **Table 1**, viz. Hyper*C*alcemia, *R*enal failure, *A*nemia, and *B*one disease, signify end-organ damage due to myeloma cell proliferation and are used for diagnosis (6). It is important to note here that SMM forms a clinically heterogeneous group, with patients with high-risk features having more aggressive disease, with a higher chance of conversion to multiple myeloma. Detecting bone disease early in these patients could lead to initiating therapy before end-organ damage occurs.

**Table 1:** Summary of the current International Myeloma Working Group (IMWG) diagnostic criteria (2019 consensus).

Summary of IMWG 2019 criteria for the diagnosis of plasma cell dyscrasias		
Multiple myeloma (MM)	Smouldering myeloma (SMM)	Monoclonal gammopathy of undetermined significance (MGUS)
Clonal bone marrow plasma cells ≥10%, *and any one* or more of the following myeloma-defining events (MDE).	Clonal bone marrow plasma cells *≥10%* (but less than 60%), or serum M protein *≥30 g/dl*	Clonal bone marrow plasma cells *less than 10%*
Colloquially referred to as the “SLiM CRAB”:		Serum M protein *less than 30 g/dl*
*S*ixty percent or more clonal bone marrow plasma cells. *Li*ght chain ratio, serum >100 (involved:uninvolved) *M*RI demonstration of > 1 Focal Lesion (of size 5 mm or more) Hypercalcemia *R*enal insufficiency *A*nemia *B*one lesions—at least one or more bone lesion on X-ray/CT/PET-CT	Absence of *all* myeloma-defining events (MDE, viz. the SLiM-CRAB)	Absence of *all* myeloma-defining events (MDE, viz. the SLiM-CRAB)

It is only recently in the 21st century that research and treatment of MM has significantly picked up pace (7) not only in the diagnostic aspects with the advent of high-resolution and functional imaging modalities but also in the therapeutic aspects with the development in myeloma-specific chemotherapeutic and targeted agents. Traditionally, MM was considered a terminal disease of the elderly, and the diagnosis required the presence of manifest end-organ damage (CRAB features). This is somewhat akin to treating a patient with breast cancer, only after distant metastases has occurred! Historically, oncologists saw no merit in treating this indolent disease because of the lack of highly effective and myeloma-specific chemotherapeutic agents. Close to four decades since the 1950s, the only drug available was melphalan: although it had some effect on the disease, its efficacy was tempered by its toxicities resulting in no clear difference in survival benefit or improvement in quality of life when given early in the disease to asymptomatic patients. We have, since, come a long way with the advent of newer agents such as lenalidomide and bortezomib, carfilzomib, and daratumumab that are more myeloma specific and less toxic, showing promising benefits (8), along with a chance of cure in those eligible for autologous bone marrow transplant. Moreover, the advent of cross-sectional anatomical imaging modalities like CT scan, magnetic resonance imaging (MRI), or functional imaging like PET scan has improved the detection rates of the lesions with far greater sensitivity and specificity than radiography alone (9, 10).

## Pathogenesis of Bone Disease in Multiple Myeloma

Destructive bone disease is the hallmark of multiple myeloma. Myeloma cells alter the bone marrow microenvironment so as to facilitate osteoclast-induced bony lysis and increased myeloma cell proliferation resulting in marrow replacing disease (11). This is effectuated by several complex signaling cascades, which involve interactions with bone marrow stromal cells and dysregulation of osteocyte function.

**Figure 1:**
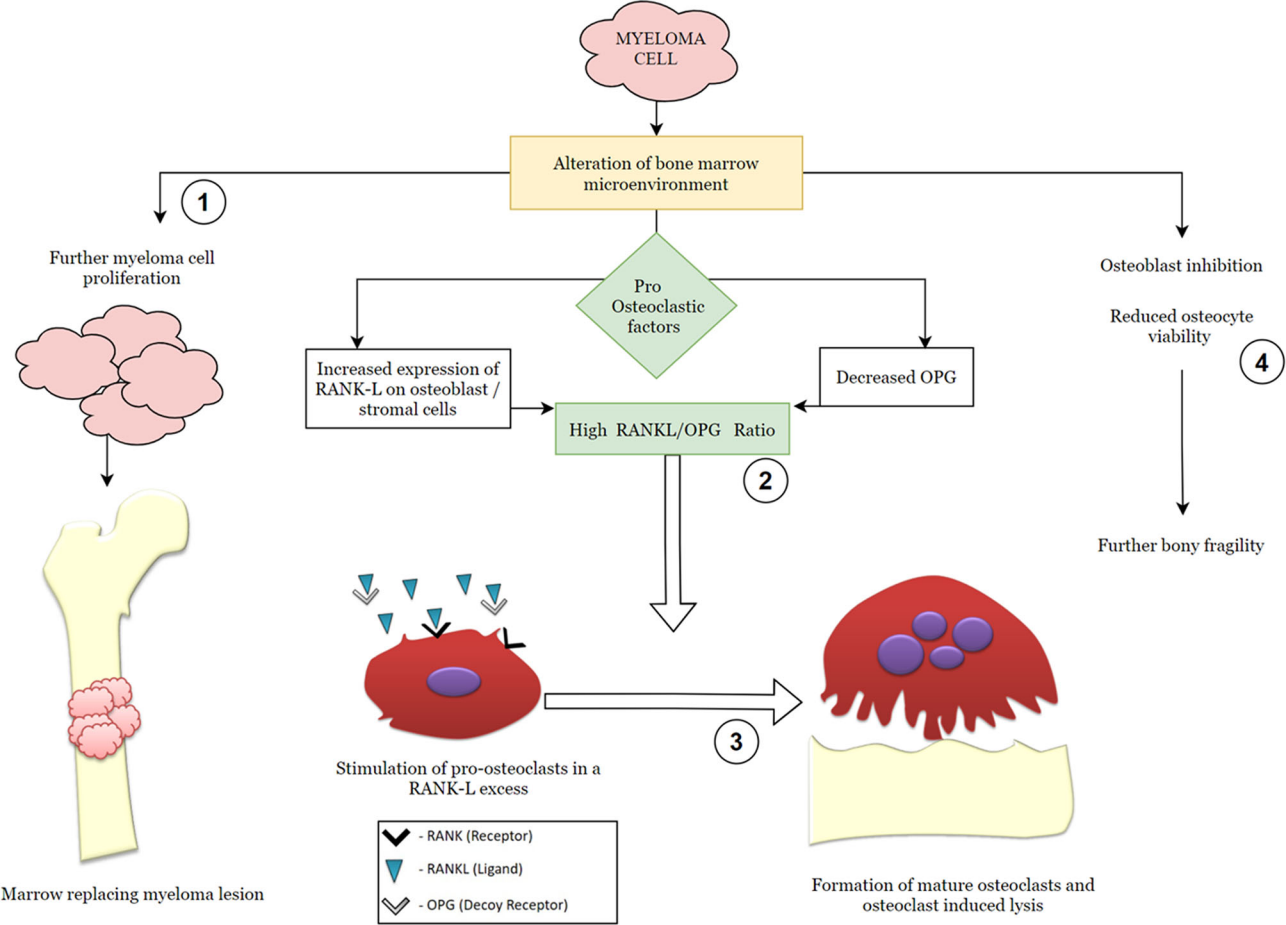
Simplified graphic demonstrating the pathophysiology of multiple myeloma with emphasis on the RANK (receptor activator of nuclear factor kappa-β)–RANK ligand–OPG (osteoprotegerin) axis. The steps in the molecular pathogenesis that serve as modulatory checkpoints for therapeutic agents have been numbered from 1 to 4 and represent targets for the following pharmacologic agents: 1—anti-neoplastic drugs (chemotherapeutic agents); 2—RANK ligand inhibitors, such as denosumab; 3—osteoclast inhibitors, such as bisphosphonates; 4—proteasome inhibitors, such as bortezomib, regulate cellular trafficking and osteocyte viability.

The key final outcome of the myeloma-cell-induced cellular changes is the receptor activator of NF-κB ligand–osteoprotegerin (RANK–RANKL–OPG) axis dysregulation (**Figure 1**). Pro-osteoclasts (osteoclast precursors) express the RANK receptor on their surface which, on binding with its ligand (the RANKL), causes osteoclast maturation. Osteoprotegerin is a decoy-soluble receptor for RANKL and preferentially binds the RANKL. It thus prevents RANK–RANKL union and inhibits osteoclast maturation. Myeloma cells elaborate on various pro-osteoclastic cytokines, known as osteoclast-activating factors (OAFs), which in turn result in an increased RANKL:OPG ratio. This results in increased osteoclast-induced bone lysis (12).

Additionally, myeloma cells directly inhibit osteoblast activity—further contributing to the state of bone fragility and osteopenia.

## Role of Imaging in Plasma Cell Dyscrasia (Multiple Myeloma)

Approximately 80% of patients with multiple myeloma show features of bone disease at presentation, with most of the remainder experiencing myeloma-related skeletal disease at some point during the course of illness. Due to the associated risk of skeletal-related events, bone disease affects the quality of life and significantly raises morbidity and mortality (9, 13, 14).

Imaging is essential for bone lesion detection, which in turn necessitates therapy initiation (15, 16). Additionally, imaging facilitates the assessment of skeletal-related events such as cord compression or compression fractures (17).

The primary objective of imaging patients with plasma cell dyscrasias is to rule out multiple myeloma by confirming the absence of any osteolytic lesions or focal lesions (FLs). This involves instituting appropriate imaging modalities and is further discussed in the text that follows.

## Imaging in a Clinically Suspected Case

### Imaging Guidelines of the International Myeloma Working Group and Evolution Over Time

**Figure 2:**
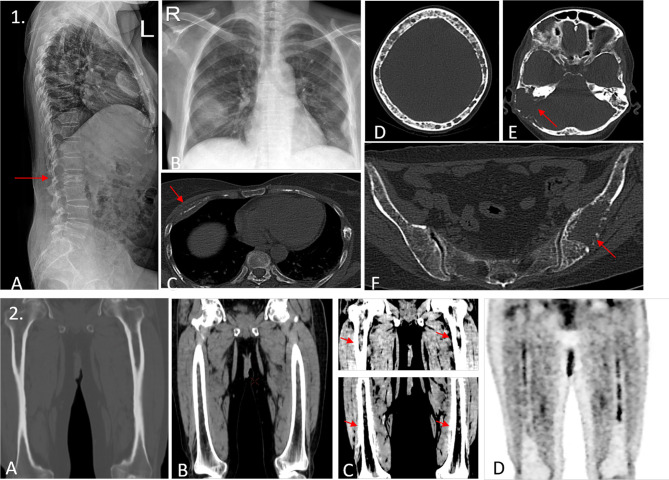
(1) A 45-year-old woman presented with L2 vertebral compression fracture as demonstrated in the lateral radiograph of the spine in image **(A)**. Blood investigations showed a positive M band, with elevated creatinine. Chest radiograph [image **(B)**] shows an ill-defined opacity in right midzone. The patient underwent a whole-body low-dose CT scan (WBLDCT), which revealed a lytic lesion with soft tissue component involving the anterior shaft of the right fourth rib [arrow, image **(C)**], which appeared consolidation-like on the radiograph. Image **(D)** the “rain-drop skull” appearance is seen with multiple variable-sized lytic lesions showing non-sclerotic borders. Image **(E)** a large lytic lesion with associated soft tissue component is seen involving the mastoid temporal bone on the right side (arrow). Image **(F)** an expansile lesion with focal areas of cortical breech is seen involving the left ilium with few other osteolytic foci scattered in the rest of the pelvis. The utility of WBLDCT lies in its ability in detecting as well as characterizing radiographically and clinically occult lesions such as the one involving the right temporal bone and the left ilium which may warrant locoregional therapy. (2) Images **(A–C)** show coronal reformatted images of both femora acquired on a WBLDCT study in a suspected case of myeloma. Note the intramedullary fat replacing hyperdense deposits best appreciated on soft tissue window settings with contrast adjustment. While these are prominent on routine soft tissue window settings as well [image **(B)**], one would entirely miss these deposits if only bone window settings were examined [image **(A)**]. Image **(D)** is a coronal reformatted MIP image from a subsequent PET/CT study that confirms the presence of these medullary deposits. Thus, careful evaluation of medullary cavities on soft tissue window settings with apt contrast adjustment can prove invaluable.

**Figure 3:**
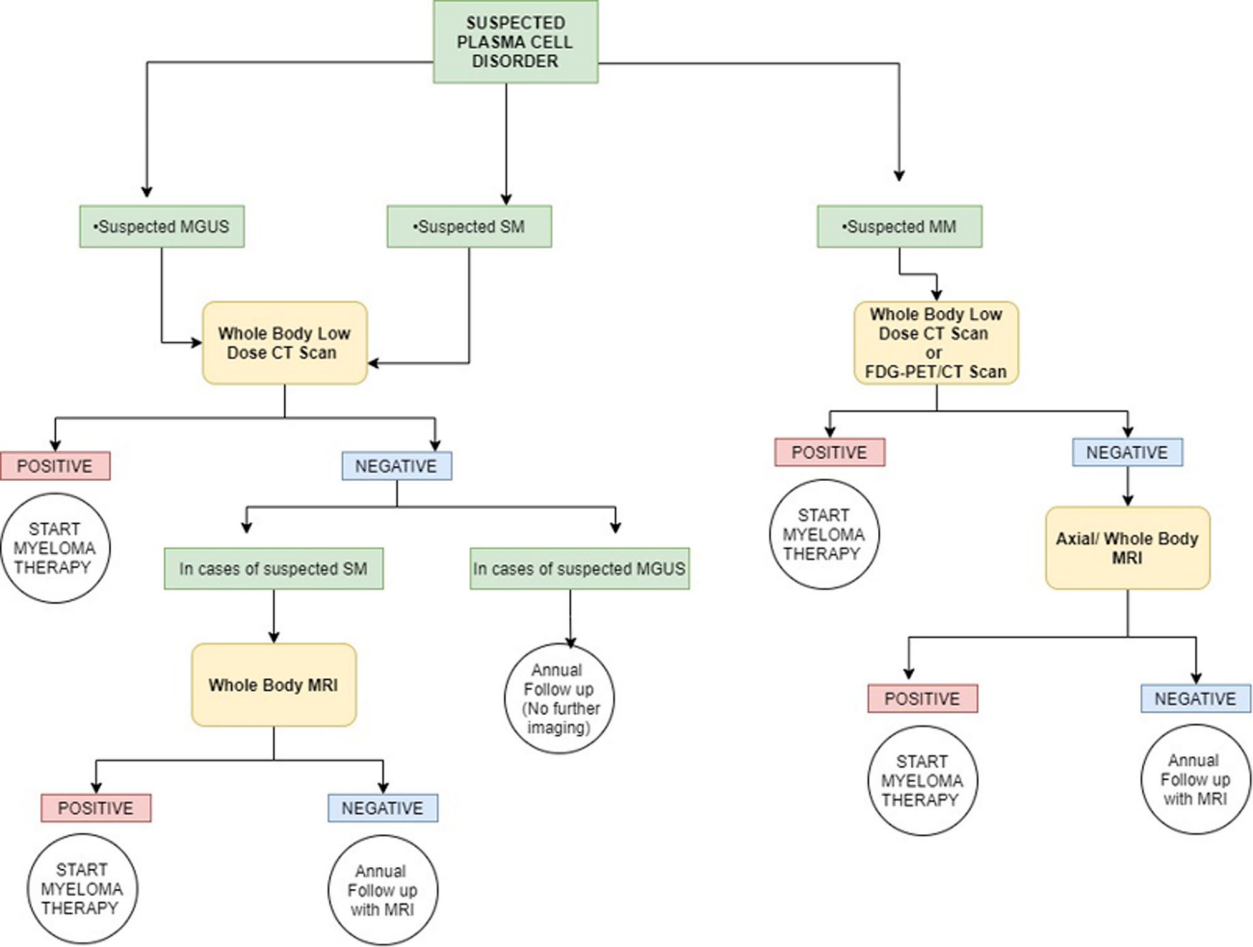
Simplified graphic representation of the imaging algorithm for initial evaluation of various plasma cell disorders adapted from the 2019 IMWG Consensus. MGUS, monoclonal gammopathy of undetermined significance; SM, smoldering multiple myeloma; MM, multiple myeloma; FDG, fluorodeoxyglucose. Overall, whole-body CT is the first imaging choice to exclude osteolytic lesions. PET/CT can be used in place of whole-body CT for suspected multiple myeloma, and it can be used in place of whole-body MRI if the MRI procedure is not feasible (or otherwise contraindicated). For a more detailed and accurate description, readers are requested to refer to the IMWG guidelines.

The International Myeloma Working Group (IMWG) in a consensus statement in 2003 for the first time defined bone disease in MM as “the presence of osteolytic bone lesions or the presence of osteoporosis with compression fractures attributable to the underlying clonal plasma cell disorder” detected on any imaging modality—radiography, CT, PET, or MRI (**Figures 2**, **3**). Studies demonstrating the benefit of initiating treatment for SMM with lytic bone lesions or asymptomatic MM and recognizing the need to identify and treat the subset of patients with SMM who have biological malignancy and are at imminent risk of progression, at a stage when it is in its “most susceptible microenvironment-dependent state,” led to the IMWG update in 2014, wherein biomarkers for MM called “myeloma defining events” were elucidated (**Table 2**) (18, 19). These updated diagnostic criteria moved multiple myeloma in line with other malignancies by removing the need for documented end-organ damage as a mandatory requirement for the definition of malignancy (20). They addressed a major drawback in terminology that prevented patients with clear-cut malignancy and at a very high risk of developing end-organ damage from receiving therapy until such damage was clinically manifested (21).

**Table 2:** Temporal evolution of the International Myeloma Working Group (IMWG) recommendations.

Year	Premise	Summary of key introductions	Key exclusions/future direction
2003	CRAB		
2014	The “SLiM CRAB”: introduction of myeloma biomarkers (myeloma-defining events, i.e., MDE)	Addition of three specific biomarkers (MDEs), i.e., 1) clonal bone marrow plasma cell percentage ≥60%; 2) involved:uninvolved serum-free light chain ratio ≥100; 3) >1 focal lesion on MRI studies (>5 mm) Presence of even one of these MDEs in the absence of CRAB is sufficient for myeloma diagnosis Single 5-mm or larger osteolytic lesion seen at PET/CT, WBLD CT, or skeletal survey: CRAB	Diffuse osteopenia and vertebral collapse no longer suffice for diagnosis Increased uptake on PET/CT alone is not adequate for the diagnosis of multiple myeloma; evidence of underlying osteolytic bone destruction is needed on the CT portion of the examination
2019	Guidelines on imaging modality according to disease stage. Detection of minimal residual disease	Validation of the role of WBLDCT as a screening tool to look for osteolytic lesions and to rule out multiple myeloma Emphasis on using FDG PET for response assessment if done at baseline	Unclear on the utility of functional MRI vs. PET/CT in response assessment—requires further validation

According to the updated criteria, the presence of two or more focal lesions that are at least 5 mm or greater in size on magnetic resonance imaging satisfies the criteria for multiple myeloma regardless of the presence of the CRAB features. The recently updated IMWG guidelines in 2019 recommended pragmatic algorithms for imaging these patients keeping in line with the evolving imaging modalities, wherein they recommend whole-body low-dose computed tomography (WBLDCT) as the first screening tool followed by whole-body MRI (WBMRI) in those patients where CT is negative.

### The Ideal Radiologic Diagnostic Technique: WBLDCT Versus WBMRI

The list of imaging modalities available in imaging suspected cases of MM, with their techniques, indications, advantages, and disadvantages, is summarized in **Table 1** and includes conventional radiography, CT scans, MRI, and PET scan, and the correct choice needs careful consideration and knowledge of the science behind the imaging modality and complete clinical picture (17).

#### Radiography Is not Recommended Unless It Is the Only Modality Available

In clinically suspected MM or high-risk SMM, the ideal imaging technique is WBMRI including a DWIBS (diffusion-weighted whole-body imaging with background body signal suppression) protocol which also addresses the functional aspect of disease to some extent and should be done in all clinically suspected patients. Screening in the general population or even a high-risk cohort is a separate issue that also involves logistics—herein, WBLDCT may be the imaging modality of choice given its universal availability, quicker examination times, short learning curve for interpretation, and relatively inexpensive cost (22).

Osteopenia defined as a generalized decrease in bone density is a common finding on X-rays or WBLDCT. This could be benign, age-related osteoporosis, wherein normal hematopoietic cells get replaced by fat, or due to malignant diffuse infiltration of bone by myeloma cells. WBMRI can help differentiate the two. Moreover, MRI is a one-stop shop for assessing complications arising due to clinically significant pain seen commonly in these patients—differentiating pathological versus benign osteoporotic fractures, cord, or nerve root compression. MRI can also identify those 3%–5% of patients with MM who are oligosecretory (23).

While the British Society for Hematology and NICE guidelines (24) prescribe WBMRI as the primary screening tool, the IMWG recommends WBLDCT for screening. Moreover, in the diagnostic workup of high-risk non-IgM multiple myeloma, if the WBLDCT is positive, the IMWG recommends that patients undergo a PET/CT to help differentiate myeloma versus metastases. The need for an additional radiation-exposing investigation can be circumvented with a WBMRI which would be potentially better able to point at other suspicious regions of primary malignancy if any, to warrant further localized investigation within the same setting, after which a biopsy can be advised. Another issue that arises from the IMWG protocol is during treatment assessment. Multiple studies have demonstrated the advantages of a functional technique like PET/CT or DWIBS MRI over purely anatomical modalities like CT, because while the lytic lesions persist for a long time (23 months to 5 years), the PET/CT reduction in uptake and the increase in apparent diffusion coefficient (ADC) can be detected early on in the course of therapy. This means that patients should ideally undergo either a PET/CT or a WBMRI at baseline for comparison after treatment even if they are positive on WBLDCT (25, 26).

### To PET or not to PET: Diagnostic Role of 18-FDG PET/CT

18-Fluorodeoxyglucose PET/CT is the gold standard for assessing treatment response. Although controversy still exists regarding the most appropriate imaging modality for diagnosing MM, some studies have demonstrated MRI to be more sensitive and specific in detecting lesions, while other studies have shown equivalent or even better specificity of PET/CT (9). However, the devil lies in the details: studies that demonstrated the superiority of PET/CT included a mixed cohort of patient population—those who were not only treatment naive but also those who received systemic treatment. PET is a better modality for assessing treatment response given its ability to detect a functional decrease in the metabolic activity of the disease (as early as 6 weeks after initiating treatment), which is much earlier than the corrections in morphological architecture that take a longer time (median time of 23 months) (27). Although 1-FDG PET is a common modality used in clinical practice, several new investigational pharmaceutical radiotracers are also being evaluated for response assessment of MM (**Supplementary Figure 1**). Until some robust evidence emerges, 18-FDG PET will continue to be used as a gold standard for treatment response assessment.

### Where to Look: Common Sites Affected in Myeloma

Typically, myeloma follows the red marrow distribution in adults, and the commonly involved sites are as follows (26, 28, 29):

- Vertebrae (most common)—66%
- Ribs—45%
- Skull—40%
- Shoulder girdle—40%
- Pelvis—30%
- Long bones—25%

## Imaging for Response Assessment

The current accepted gold standard for response assessment of MM is PET/CT (30). Even in patients whose bone marrow, serum, and urine results show complete response, there may still be a residual disease because bone marrow involvement in MM can be patchy, increasing the likelihood of a false-negative result if one were to rely only on trephine biopsy from a single site (25, 31, 32). Furthermore, up to 20% of patients may present with only extramedullary disease at relapse (33). Hence, PET/CT is advised in follow-up and assessing functional disease activity at an interval of months in all patients of MM on therapy (34–36). WBMRI is excellent in the evaluation of disease progression; however, for assessment of complete response or objective response, MRI is not adequate. Development of a sclerotic margin, new internal fat attenuation, and regrowth of cortical bone are CT features frequently seen with positive treatment response. Despite this, CT has a relatively limited role in treatment response evaluation, especially as compared with PET/CT and MRI (37, 38).

## Complications Arising During the Course of Treatment: Which Imaging Modality to Use?

**Figure 4:**
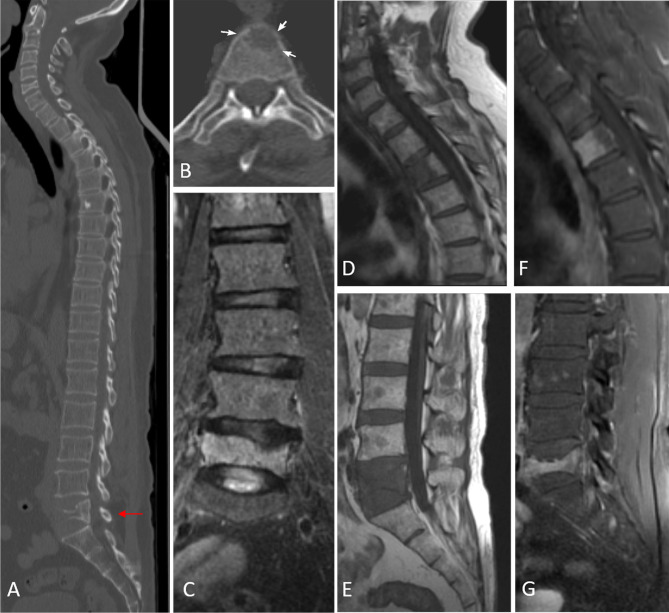
Image **(A)** sagittal CT image in bone window demonstrating vertebral compression fracture with >75% height reduction (arrow). Image **(B)** shows a lytic lesion involving the C7 vertebral body. MRI images confirm pathological L5 vertebral fracture with the concave posterior bulge of the vertebra, STIR hyperintensity seen in coronal STIR image of the lumbar spine [image **(C)**], T1 hypointensity [image **(E)**], and post-contrast enhancement [image **(G)**]. The lytic cervical vertebral lesion seen on CT in image **(B)**, is well appreciated in images **(D, F)**. Additionally, MRI reveals a variegated pattern with multiple punctate foci of altered signal intensity involving the marrow.

Patients may develop severe bone pain/backache at any point during or after treatment, and this is most commonly due to fractures (39). MRI is advised in imaging complications that arise during the course of therapy because of its ability to distinguish benign versus malignant fractures (40) (**Figure 4**). Not all fractures that occur in MM patients on treatment are due to disease progression. MM is a disease of the elderly, who are prone to osteoporotic degeneration-related fractures. Also, as the myeloma cells respond to treatment and shrink in size, they leave behind a dead space of weakened bone that are picked up by MRI (7). On the other hand, myeloma cells can develop resistance to chemotherapy and cause new-onset lesions with associated fracture. PET/CT, on the other hand, is not specific enough to differentiate benign from malignant fractures as the metabolic uptake is high in both cases due to acute inflammatory changes; hence, WBMRI with DWIBS is the best available modality to help distinguish the two (34, 41) (**Supplementary Figure 1**).

### Differentiating Red Marrow Reconversion From Disease

Patients with myeloma receive regular g-CSF injections to treat anemia during the course of their treatment. As the tumor burden decreases as a response to effective chemotherapy, the marrow slowly starts getting replaced by hematopoietic marrow which can also appear to be hypointense to the adjacent vertebral disc on T1MRI (42). In contrast to the orderly fashion of normal marrow conversion, reconversion is a patchy and an asymmetrical process (43) and this leads to a difficult clinical question in the post-treatment setting wherein abnormal marrow signal could represent poor responding/relapsed disease versus red marrow reconversion (30).

**Figure 5:**
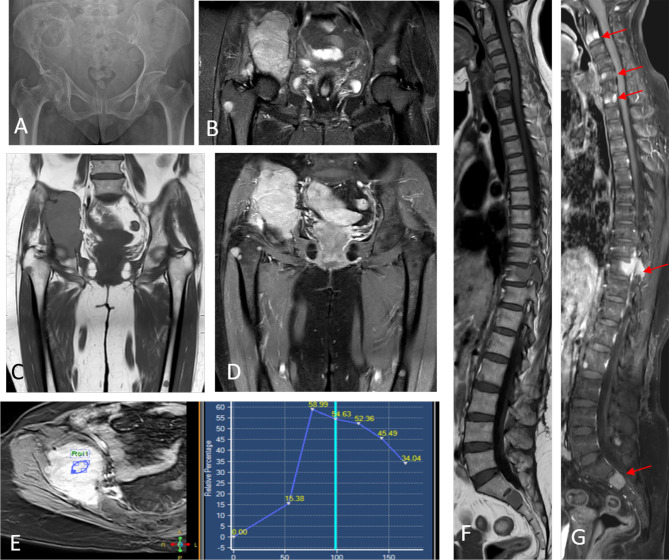
Radiograph of the pelvis [image **(A)**] shows a large lytic lesion with associated soft tissue component involving the right iliac bone. Image **(B)** shows a coronal STIR image that further delineates the right iliac mass and additionally demonstrates other STIR hyperintense foci in the contralateral left hip as well as the right proximal femur. Coronal pre-contrast T1W [image **(C)**] and contrast-enhanced T1W [image **(D)**] series show intense post-contrast enhancement within the right iliac mass and multiple other enhancing marrow foci scattered in the pelvis and both femora. A dynamic contrast enhancement analysis [image **(E)**] revealed a type III kinetic curve demonstrating rapid enhancement and washout within the lesion. Additional MRI imaging of the spine shows multiple T1 hypointense [image **(F)**] marrow lesions that show intense post-contrast enhancement [arrows in image **(G)**]. Biopsy from the right iliac mass revealed plasma cell proliferation on histopathology, with overall findings consistent with multiple myeloma.

Normal adult red marrow is comprised of 40%–60% lipids and yellow marrow contains up to 80% lipids. Hence, a Dixon sequence with in- and out-of-phase images would demonstrate a greater than 20% drop in signal in the case of normal marrow; this is not usually seen in myeloma or metastasis (42). Also, diffusion-weighted imaging (DWI) can help differentiate the two as ADC values are high in diffuse bone marrow involvement patterns and low in red marrow reconversion (43). Finally, dynamic contrast-enhanced sequences (DCE-MRI) with the same principles as in other organ systems may help differentiate the two as normal marrow shows slow progressive enhancement on time intensity curves while myeloma shows rapid uptake with rapid washout or plateauing (32) (**Figure 5**).

A final concern regards bone marrow iron overload which could happen as a sequel to repeated blood transfusions, and this leads to decreased signal intensity on all MRI sequences, including DW imaging and ADC imaging (25, 44).

## Modality Summary: Conventional Radiography

- Principle: Abnormal myeloma cell proliferation, resulting in mechanical burden that compromises the skeleton by displacing and eroding the bony trabecular tissue manifesting as osteolytic lesions (25, 35) (**Table 3**).
- Technique: Skeletal survey: AP and lateral views of the skull, chest, spine, and pelvis with both hips, and long bones. Total body dose 1.7–2.4 mSv (26).
- Imaging findings: Osteolytic lesions involving the axial skeleton and calvarium, with generalized osteopenia and vertebral compression fractures. “Raindrop skull” refers to the typical appearance of small punched-out lytic lesions studding the calvarium and can also be observed on reconstructed CT images.
- Limitations: Detection of osteolysis is possible only when 50%–75% of cancellous bone has been replaced, thus very poor sensitivity and very high false-negative numbers. The sensitivity is even lower for the axial skeleton. This is presently not recommended in the evaluation of plasma cell dyscrasia unless all of the other imaging modalities are unavailable.

**Table 3:** Summary of technique, principles, and utility of various imaging modalities for plasma cell disorders.

Modality	Protocol	Indication	Principle of imaging	Findings	Advantages	Disadvantages	Recommendation
Radiography	Skeletal survey: AP and lateral views of the skull, chest, spine, PBH, long bones	Diagnosis and complication assessment	Proliferation of abnormal plasma cells creates a mechanical burden that compromises the skeleton by displacing and eroding bony trabecular tissue	Lytic areas of uniform size and endosteal scalloping in the skull, axial skeleton	• Available easily and commonly used modality across the world • Economical	• Difficulty in positioning patients with severe pain • Low sensitivity (30%–50%) (45); even lower in axial skeleton (32) Needs at least 50% trabecular destruction to manifest	Not recommended, except if no other modality available
	Total body dose 1.7–2.4 mSv		Plasma cells produce an osteoclast stimulating factor-osteolytic lesions and also leads to inhibition of osteoblasts	Differentiator from metastases: predilection for sites such as mandible, clavicle, glenoid, vertebral body (rather than posterior elements)			
				Diffuse osteopenia Vertebral collapse/fractures			
Technetium bone scan		Diagnosis or follow-up					Not recommended
Whole-body low-dose CT (WBLDCT)	120 kV, 40–50 mAs, slice thickness 2 mm	Diagnosis and imaging complications: vertebral collapse/fractures	Same as radiography, only much more sensitive (can detect even 5% trabecular destruction) (19) and specific	Similar to X-rays—lytic lesions, diffuse osteopenia, endosteal scalloping, neoplastic and osteoporotic fractures, cortical disruption, and extraosseous involvement	• Good PPV 94% (46) • Sensitivity of 69.6% and a specificity of 90.9% • Less expensive than other cross-sectional imaging modalities • Quick: acquisition time is 40–60 s • Excellent interobserver correlation • Low radiation dose protocol: 3.2–4.8 mSv	• Poor NPV = 58% • Cannot evaluate marrow infiltration	Best screening tool—ESMO and EMN
	No contrast needed						
	Hands overhead to reduce beam hardening on the spine						
MRI spine	Sagittal T1-weighted, STIR; slice thickness 4 mm, 512 × 512 matrix size	Diagnosis or complication assessment	Marrow infiltration by abnormal cells and be detected before frank lytic lesions appear	Five patterns: apparently normal bone marrow, diffuse involvement, focal involvement, combined diffuse and focal involvement, and variegated, or salt and pepper. T1 most important sequence: to look for hypointense lesions replacing normal fatty marrow of vertebrae	• Excellent sensitivity and specificity: 68% to 100% and 83% to 100%, respectively (33, 37)	• 10% of patients have only appendicular involvement: false negative in these (34)	Reasonable accuracy of 90% where WBMRI is not available (25)
Whole-body MRI (WBMRI)	Coronal sequences T1-weighted, STIR, axial DW, 5 mm slice, 512 × 512 DWIBS axial *b* = 50 and 800 s/mm^2^	Diagnosis or complication assessment	Similar as above	Same as above. MM lesions appear as areas of increased diffusivity compared with low diffusion in normal background marrow	DWI most sensitive sequence, changes in T1 occur later (22, 23)	• Long time, technical expertise needed • Expensive and more cumbersome for the patient • Not as effective as PET/CT in response assessment	Gold standard (26) for: Diagnosis Complication assessment Staging solitary plasmacytoma
					Sensitivity (68% to 100%) and specificity (83% to 100%) (33)		
Positron emission tomography (PET/CT)	Intravenous dose of about 13.7 mCI of 18F-FDG; PET images from the skull to the femora, including the upper limbs after a 1-h delay	Treatment response assessment Diagnosis	Metabolically active tumor cells show active FDG uptake	Raised SUV with underlying lytic lesion	• Functional plus morphological information • Shows functional response to treatment much before morphological changes manifest on other modalities • Sensitivity (59% to 100%) and specificity (75% to 82%) (34)	• Less sensitive than MRI especially in patients with diffuse marrow involvement (26) • FP: inflammation/post-biopsy • FN: hexokinase 2 deficiency	Gold standard for response assessment (35, 38, 42) Also helpful in prognostication
	Total body dose of about 21.64 ± 5.20 mSv						

## Modality Summary: Whole-Body Low-Dose Computed Tomography

- Principle: The principle of WBLDCT is the same as skeletal radiography—abnormal myeloma cell proliferation—resulting in mechanical burden that compromises the skeleton by displacing and eroding bony trabecular tissue manifesting as osteolytic lesions. However, spatial resolution and anatomical depiction are far superior owing to cross-sectional acquisition (17, 47).
- Technique: Acquisition on a 16-slice or higher multidetector CT with tube voltage/time–current parameters of 120 kV/50–70 mAs; slice thickness 2 mm. Scan coverage from the skull vault to the proximal tibial metaphysis, with collimation of 0.5–1.5 mm. No contrast needed. Total effective dose of about 4.1–7.5 mSv (23, 48).
- Utility in diagnosis: Recommended initial imaging modality to identify and assess the extent of osteolytic lesions in high-risk MGUS, SMM, and MM as per the IMWG recommendations (20). WBLDCT has replaced conventional radiography, owing to its greater sensitivity (of about 69%) than conventional radiography with the ability to detect bony lesions with minimal bony destruction (up to 5% bony destruction). High specificity (of about 90%) (49) and short scan acquisition times led to its widespread use as an initial diagnostic modality for suspected plasma cell dyscrasias. Other advantages are listed in **Table 3**.
- Utility in response assessment or relapse:

WBLCT is not considered an ideal modality to assess therapy response. The presence of new lesions on follow-up CT imaging represents progression. Treatment response may be seen in the form of intralesional or peripheral rim sclerosis and reduction of the lytic component. Development of fat density within the lesion may be observed occasionally (50). However, PET/CT is the ideal imaging modality for response assessment.

E) Interpretation of findings:

- Osteolytic myeloma lesions: Osteolytic lesions without sclerotic borders that are at least 5 mm in diameter are sufficient to meet the CRAB criteria. These may be associated with endosteal scalloping, cortical breach, and frank extraosseous soft tissue components (paramedullary disease). The associated cord compromise when present must be further evaluated with an MRI.
- Medullary disease: Medullary myeloma deposits in the femora and humeri can be seen as hyperattenuating lesions in the background of yellow marrow.
- Diffuse osteopenia, with osteoporotic and/or neoplastic fractures, including vertebral compression fractures.

F) Additional tips during WBLDCT scanning:

- Patient hands to be placed above the head during acquisition with minimal bending at the elbow to enable coverage of the humeri without significant beam hardening artifacts at the level of the vertebra. If the arms are placed by the sides, ensure they do not touch the scanning table and are kept elevated or in front of the body.
- Use of sharp, high-frequency kernel (bone algorithm) and smooth (soft tissue) reconstruction to be evaluated systematically, with appropriate use of multiplanar reconstruction (MPR) (51). MPR with sagittal reformations of the spine and coronal reconstructions of the appendicular skeleton are invaluable in having a good quality scan.
- Lesion density must be measured on soft tissue reconstructions rather than soft tissue windows of sharp kernel bone algorithm images to avoid spurious HU values.
- Fat-containing hypodense lesions in treatment-naive cases effectively rule out myelomatous disease and may represent intraosseous lipomas, small hemangiomas (52), focal yellow marrow islands, or fatty Modic changes (53, 54). Apt density measurement to demonstrate an HU of up to - 100 should be employed.

G) Limitations: Not sensitive to marrow lesions and does not provide metabolic/functional information. Thus, limited by a low negative predictive value and ought to be supplemented with PET/CT or MRI in order to rule out FLs.

## Modality Summary: Magnetic Resonance Imaging

- Principle: Detection of marrow infiltration by abnormal myeloma cells making use of signal intensity alterations that result from fat replacement (25).
- Technique: WBMRI protocol: whole body: coronal T1-weighted sequences, STIR, axial DW, DWIBS axial sequences. Dynamic contrast-enhanced sequences are optional.

Whole spine and pelvis: sagittal T1-weighted (preferably Dixon), STIR sequences of the spine with coronal T1-weighted and STIR images of the pelvis (46).

- C) Utility in diagnosis: MRI is the imaging gold standard for marrow involvement in myeloma (25), with detection of a higher number of FLs as well as diffuse marrow lesions compared with PET/CT. The sensitivity and specificity of WBMRI are in the range of 68% to 100% and 83% to 100%, respectively (5, 49). In cases where WBMRI is unavailable, axial MRI scanning of the spine and pelvis may be substituted as it has been shown to detect about 90% of FLs. On confirmation of the absence of osteolytic lesions on WBLDCT, WBMRI/axial MRI scanning is the modality of choice to rule out focal lesions in the cases of SMM and high-risk MGUS (19, 21). MRI can also accurately illustrate the spinal cord and/or nerve root compression for surgical intervention or radiation therapy.
- D) Utility in response assessment and prognostic role: The presence of more than seven FLs and diffuse pattern of involvement are harbingers of inferior survival. PET/CT is superior to conventional MRI in terms of lesion viability detection, with conventional MRI demonstrating a sensitivity of 64% for lesions that are in remission (20). However, preliminary reports with the use of DW-MRI are promising and indicate a strong correlation between increasing ADC values and remission, warranting further studies to validate these results. MRI features of response to therapy may include the appearance of a T1 hyperintense halo at the periphery of FLs with an increase in ADC values. Previously diffuse patterns may convert to variegated or focal lesions (55).

MRI also serves as an effective tool to image complications such as pathological fractures, cord compression, and other skeletal-related events. Additionally, it enables differentiation between normal and abnormal marrow (56).

**Figure 6:**
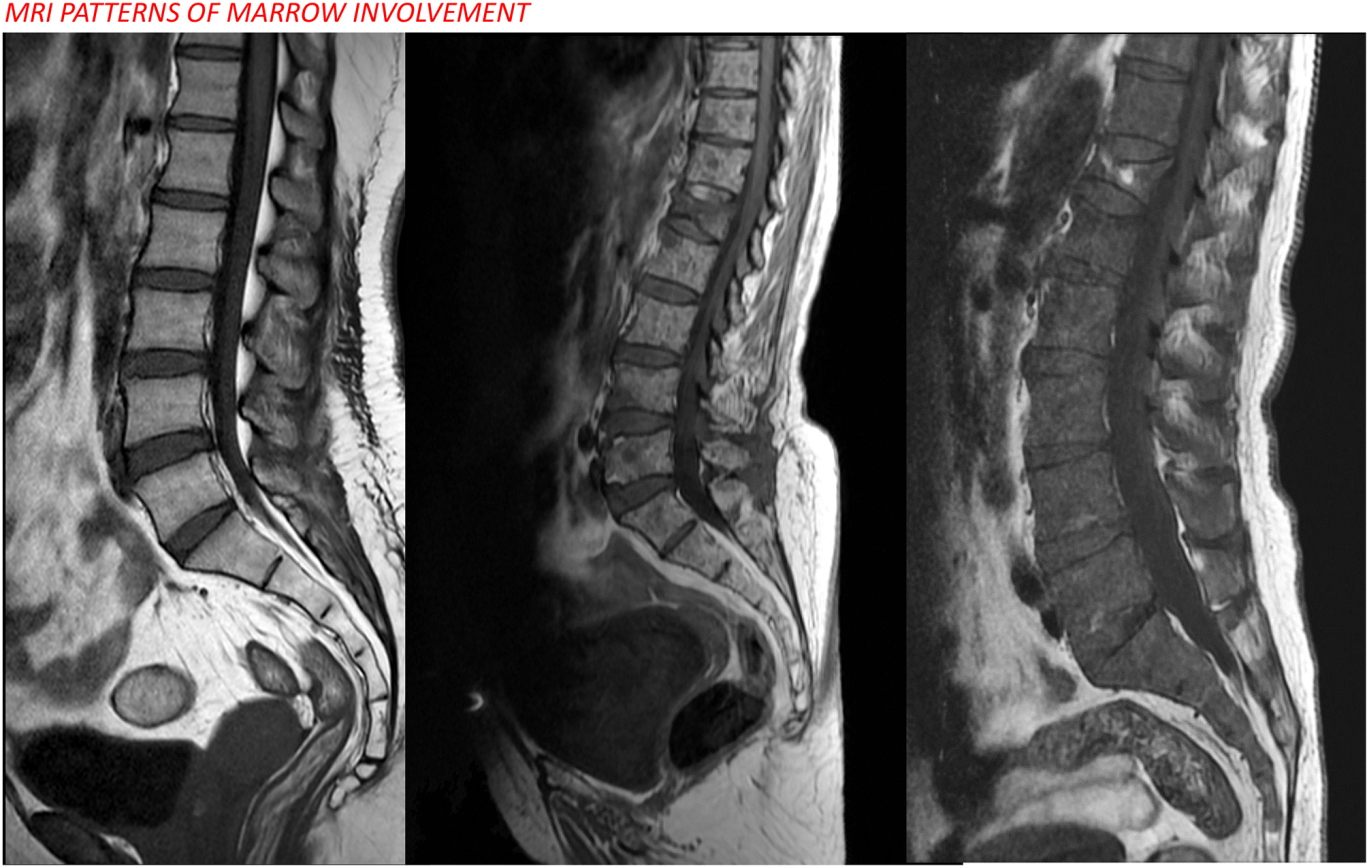
MRI patterns of marrow involvement in multiple myeloma showing normal, focal, and diffuse patterns from left to right.

- E) Interpretation of findings: Five patterns of bone marrow involvement are observed: apparently normal bone marrow, diffuse involvement, focal involvement, combined diffuse and focal involvement, and variegated/salt-and-pepper appearance (25) (**Figure 6**).

The presence of at least two focal lesions measuring more than 5 mm is classified as a myeloma-defining event (57). Diffuse and variegated/salt-and-pepper patterns are not diagnostic for multiple myeloma but may have prognostic implications (58).

**Figure 7:**
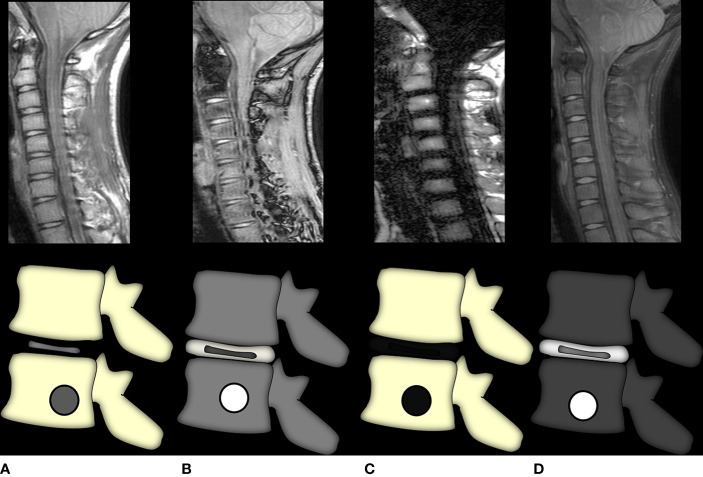
Graphic representation of the Dixon method. Image **(A)** is the in-phase image, akin to a regular T1-weighted image where marrow fat is hyperintense and any marrow replacing lesion (or red marrow) would be seen as T1 intermediate to hypointense signal intensity. Image **(B)** shows the out-of-phase sequence wherein all voxels containing microscopic fat would show a dropout of signal and appear hypointense with India ink artifacts at water–fat interfaces. Thus, lesions that do not suppress this sequence are non-fat-containing. Images **(C, D)** show fat-only and water-only sequences, respectively, which further selectively demonstrate fat and non-fat signal intensities, respectively. The water-only sequence brings about the most selective fat suppression and shows the greatest lesion conspicuity.

- Role of conventional MRI sequences: T1-weighted images are the most important sequence and the authors suggest taking the Dixon protocol which gives T1-weighted fat-only, water-only, in-phase, and out-of-phase images all at once (41) (**Figure 7**). The spine provides us with an internal control wherein all normal vertebra in adults show a signal intensity that is higher than that of the adjacent disc/intervertebral muscles (5). A decrease in the marrow signal intensity could mean marrow infiltration, and the pattern can be diffuse, patchy, focal, variegated, or mixed. However, not all marrow infiltration is a disease. Diffuse/patchy red marrow reconversion can also cause a similar appearance, and the radiologist needs to be aware of how to differentiate the two.
- Diffusion-weighted imaging of the spine: The single-shot echo-planar imaging (SS-EPI) technique is the most commonly used sequence in DWI. The signal obtained reflects the water content of the tissue, which is influenced by both perfusion and diffusion. A diffusion-weighted image is created by applying diffusion-sensitizing gradients to a T2-weighted image, where the parameters of the sensitizing gradient are determined by the *b*-value. With a *b*-value of 0, the image appears as a T2-weighted image and a progressive increase in the *b*-value begins to suppress the perfusion effect, with only highly cellular tissues remaining bright at high *b*-values (42, 45). A hyperintense signal on DWI corresponds to an area where water motion is restricted and is not able to move out of the image plane (45).

DWI of the bone is uniquely different from the pattern seen in all other organ systems—normal bone marrow shows restricted diffusion with low ADC values, whereas disease (metastases/myeloma) leads to a facilitated diffusion with a progressive increase in ADC values. ADCs of normal bone marrow are very low (range, 0.2–0.5 × 10^−3^ mm^2^/s), mainly due to low proton density and the abundance of marrow fat, which acts as a physical barrier to the free diffusivity of water molecules (59). Moreover, bone trabeculae and decreased vascularity also contribute to the restricted diffusion of normal marrow (25, 60). Any pathologic process, including focal or diffuse myelomatous infiltration, which replaces normal marrow will, therefore, appear as an area of increased diffusivity (i.e., with higher ADCs), compared with the restricted diffusion of normal marrow (59). Normal, focal, and diffuse MR imaging patterns of the bone marrow in patients with MM have distinct ranges of ADCs on diffusion-weighted images (mean ± standard deviation, 0.360 × 10^−3^ mm). Koutoulidis et al. found that a bone marrow ADC greater than 0.548 × 10^−3^ mm^2^/s had 100% sensitivity and 98% specificity for the diagnosis of a diffuse MR imaging pattern (vs. a normal MR imaging pattern) in patients with MM, whereas a value greater than 0.597 × 10^−3^ mm^2^/s showed 96% sensitivity and 100% specificity (59).

In addition to the *b*-values of 0 and 400, an additional high *b*-value of 800 is also needed in imaging the bone in MM (44). The value of acquiring additional high *b*-values is that increasing diffusion weighting reduces false-positive hyperintense osteoporotic fractures or make hypointensity more obvious in osteoporotic fractures so as to differentiate acute benign osteoporotic fractures from spontaneous vertebral compression fractures caused by a malignant lesion, since both types of fracture exhibit similar signal changes on routine MR images (61, 62).

- iii) Diffusion-weighted whole-body imaging with background body signal suppression: DWIBS is a free-breathing sequence wherein multiple thin slice axial sections of the whole body are acquired. It relies on the relatively unchanged “incoherent” motion within a voxel during respiration where the “coherent” motion is affected. It is the incoherent motion of the water molecules that determines diffusivity (45, 63, 64).

Although respiratory gating may improve the image resolution, it is an extremely time-consuming acquisition even for limited coverage, let alone WBMRI. Thus, it is more advantageous to volumetrically acquire more data in a free-breathing state to increase the number of signal averages and generate higher SNR. High-quality MPR can be performed owing to the thin slice acquisition (65). A STIR (short tau inversion recovery) sequence is the commonly employed prepulse fat-saturating sequence that is combined with DWIBS to achieve uniform fat suppression (42). *b*-values generally range from 800 to 1,000 s/mm^2^. Post-processing involves inversion of gray scale, followed by generation of MIP and reformatted sagittal/coronal plane images. Additionally, volume rendering and fusion with other sequences can also be performed (66).

The fast speed of acquisition, whole-body coverage, high lesion conspicuity with suppression of background signals, and the inherent detection of diffusion restriction make DWIBS a highly useful tool in both detection and response assessment of myeloma lesions (25).

The pitfalls of this sequence include artifacts due to inhomogeneous fat suppression and aberrant interpretation due to varying marrow fat content with age. Although DWIBS MRI is highly sensitive, the specificity of findings picked up by DWIBS needs to be validated and remains an area of possible false positives (13).

- iv) Dynamic Contrast-Enhanced MRI: The angiogenic switch theory is the basis of the utilization of dynamic contrast-enhanced images in multiple myeloma. DCE-MRI involves the multiphase dynamic acquisition of T1-weighted fat-saturated images before, during, and after contrast administration followed by perfusion assessment (32, 67). Most commonly, the time–intensity curve (TIC) is used to qualitatively assess the perfusion characteristics (52, 68). The normal enhancement curve of the marrow begins as a low maximal enhancement followed by a plateau or minimal washout. Due to the neoangiogenesis brought about by myeloma cell-induced alteration of the marrow milieu, a rapid upstroke followed by rapid washout is seen in cases of multiple myeloma (59).

## Modality Summary: PET/CT

- Principle: FDG PET/CT is a whole-body imaging technique that provides morphological as well as functional assessment of metabolically active disease burden (69). The 18F-FDG study relies on increased glucose demand in the rapidly proliferating tumor cells. Overexpression of the surface transporter GLUT in tumor cells results in the trapping of the glucose analog (18F-FDG) within these cells (35). As 18F-FDG cannot be degraded further, the radioactive moiety accumulates within these cells and is detected as FDG avidity (70, 71).
- Technique: Intravenous dose of about 13.7 mCI of 18F-FDG; PET images from the skull to the femora, including the upper limbs after a 1-h delay (25).
- Utility in diagnosis: Owing to its whole-body coverage, 18F-FDG PET/CT is superior to MRI for the detection of extramedullary disease (72). The accuracy for bone lesion detection may be comparable to whole-body MRI (1), barring diffusely hypermetabolic marrow or the skull wherein the normal hypermetabolic activity of the brain may mask the underlying tumor (73). In cases of whole-body X-ray negativity and unavailability of whole-body MRI, 18F-FDG PET/CT can be a useful tool to look for active disease (71, 74).
- Utility in response assessment and as a prognostic indicator: 18F-FDG PET/CT is the gold standard for response to therapy in MM. Complete suppression of FDG avidity on post-therapy scans confers increased overall survival and serves as a good prognostic marker (11). The persistence of severe 18F-FDG uptake in the form of the number of focal lesions, SUVmax, and the presence of extramedullary disease is a predictor of poor outcome (75).
- Limitations: Poor sensitivity in the detection of skull or diffuse marrow infiltration due to masking of the tumor activity in these metabolically active sites is an important drawback of PET/CT (76, 77). False-negative results may be obtained in patients with hexokinase deficiency. The lack of established criteria for FDG PET/CT image interpretation results in wide interobserver variation (69).
- Emerging radiotracers in the diagnosis and response assessment: 18F-FDG has limitations as a radio-pharmaceutical in the detection of diffuse bone marrow infiltration leading to false-negative results in a significant proportion of patients. Thus, there is new ongoing research in the development of several alternative PET tracers, some of which show preliminary promising results regarding MM detection (78) (**Table 4**).

**Table 4:** Summary of various upcoming radiotracer-based molecular imaging techniques.

Radiotracer	Principle	Utility in comparison with 18F-FDG	Limitations
18F/11C Choline	Choline: substrate for cell membrane biosynthesis, hence increased uptake by proliferating cells with high membrane turnover	Higher detection of focal lesions compared with 18F-FDG	Masking of marrow/liver lesions—owing to physiologically raised choline uptake Very short half-life: need for on-site cyclotron Radiotracer synthesis technically challenging
11C Acetate	Acetate: precursor to acetyl-CoA synthase (lipid synthesis key enzyme). Proliferating plasma cells—increased lipid synthesis; thus, increased uptake	Higher detection rate for both diffuse and focal myeloma lesions than 18F-FDG Role in response assessment: significant SUVmax differences noted in pre- and post-therapy states	Radiotracer synthesis technically challenging Need for on-site cyclotron
11C Methionine	Methionine: amino acid PET tracer; rapidly incorporated by proliferating plasma cells into immunoglobulins	Higher detection of focal and diffuse lesions compared with 18F-FDG Better detection of skull lesions (low physiological uptake by the brain)	Masking of marrow/liver lesions—owing to physiologically raised methionine uptake Radiotracer synthesis technically challenging
68Ga-Pentixafor	68Ga-Pentixafor: strongly binds chemokine receptor 4 (CXCR4), a G-protein-coupled chemokine receptor. This receptor is upregulated in MM and mediates various steps in MM pathogenesis	Theragnostic potential May have better detection rates than 18F-FDG Prognostic biomarker: as it correlates with end-organ damage and other lab parameters	Radiotracer synthesis technically challenging
89Zr-Daratumumab	Daratumumab: anti-CD38 monoclonal antibody. CD38—overexpressed by myeloma cells	Early stages of development Theragnostic role	Phase I trial—limited data
18F-Sodium fluoride (18F-NaF)	NaF: marker of osteoblastic activity	Poor sensitivity owing to the suppressed osteoblastic action in MM Poor specificity—uptake in any focus of bone reconstruction	Poor detection rate
18F-Fluorothymidine (18F-FLT)	Fluorothymidine: thymidine kinase acts upon this substrate to produce high-energy phosphates that are trapped intracellularly. High thymidine kinase activity in rapidly proliferating cells responsible for increased uptake	Inferior to FDG PET in the detection of lesions	Poor detection rate

## Radiologist-Initiated Suspicion

Backache is a common complaint in the elderly and imaging is performed to detect the 5% of patients who may have an underlying serious pathology, and less than 1% of these patients will have myeloma (5, 79). Looking for marrow signal abnormality on T1W spin-echo sequences is the most important, and herein, we are blessed with an internal control where, in the normal adult spine, the vertebral body is always brighter than the adjacent disc (43). When this relationship is inversed, the possibility of a marrow infiltrative disorder or marrow reconversion needs to be considered and some additional sequences are advised to help differentiate the two (80, 81).

STIR is a standard sequence included universally in MR spine protocols and helps detect marrow edema or infiltration. Post-contrast studies demonstrating enhancement of marrow, especially focal lesions, are also helpful because normal marrow in adults shows little or no enhancement. In the presence of the above, radiologists must advise further clinical and hematological workup for a marrow infiltrative disorder (82).

Osteoporosis-related degenerative spines can show MODIC changes around the end plates (53). Marrow reconversion can also begin around the end plates in the form of band-like signal abnormality as described by Stevens et al. Moreover, MM can also present in up to five different patterns as described below. The diagnosis is easy when there is diffuse marrow involvement. The problem becomes compounded when there is a patchy signal abnormality in the spine on T1W images. This is because red marrow in the normal lumbar spine also persists as a patchy area. However, there are predictable albeit several ways of red marrow reconversion as described by Berg et al. (83, 84). After successful bone marrow transplantation, hematopoietically active stem cells repopulate the bone marrow in a predictable pattern. A few weeks after the transplant, a band-like zone becomes visible in the periphery of the vertebral centrum, particularly beneath the end plates. This zone exhibits an intermediate signal on both T1w and T2w images, similar to red marrow. The band enlarges over time and is easily distinguished from central fat. Histologically, this band of intermediate signal is composed of hematopoietically active cells (85). The time course is variable but occurs in almost all patients within the first 90 days. Moulopoulos reported that in their experience the marrow repopulation gradually becomes more homogeneous over time (86).

As a final note, to promote standardization and diminish variations in the acquisition, interpretation, and reporting of whole-body MRI in myeloma and allow response assessment, the IMWG and the NICE UK group together developed the Myeloma Response Assessment and Diagnosis System (MY-RADS) (14, 86) (**Tables 5** and **6**). A simplified structured reporting format adapted from the MY-RADS group is available in **Supplementary Figure 2** as a reference guide for radiologists.

**Table 5:** Structured reporting format in a case of multiple myeloma.

**• Clinical indication:** Diagnostic workup/Complication assessment/ Response assessment.	
**• History:** Complaints, treatment received, G-CSF, h/o vertebroplasty, h/o transplant.	
**• Technique:** WB-MRI/Whole spine protocol to be clearly mentioned.	
**• **Compared with Previous scans dated_ (If Present).	
**FINDINGS:**	
**(I) Bone evaluation:**	**(II) Extramedullary Sites**
** Total number of bone lesions: 0, 1, 2-7, >7**	
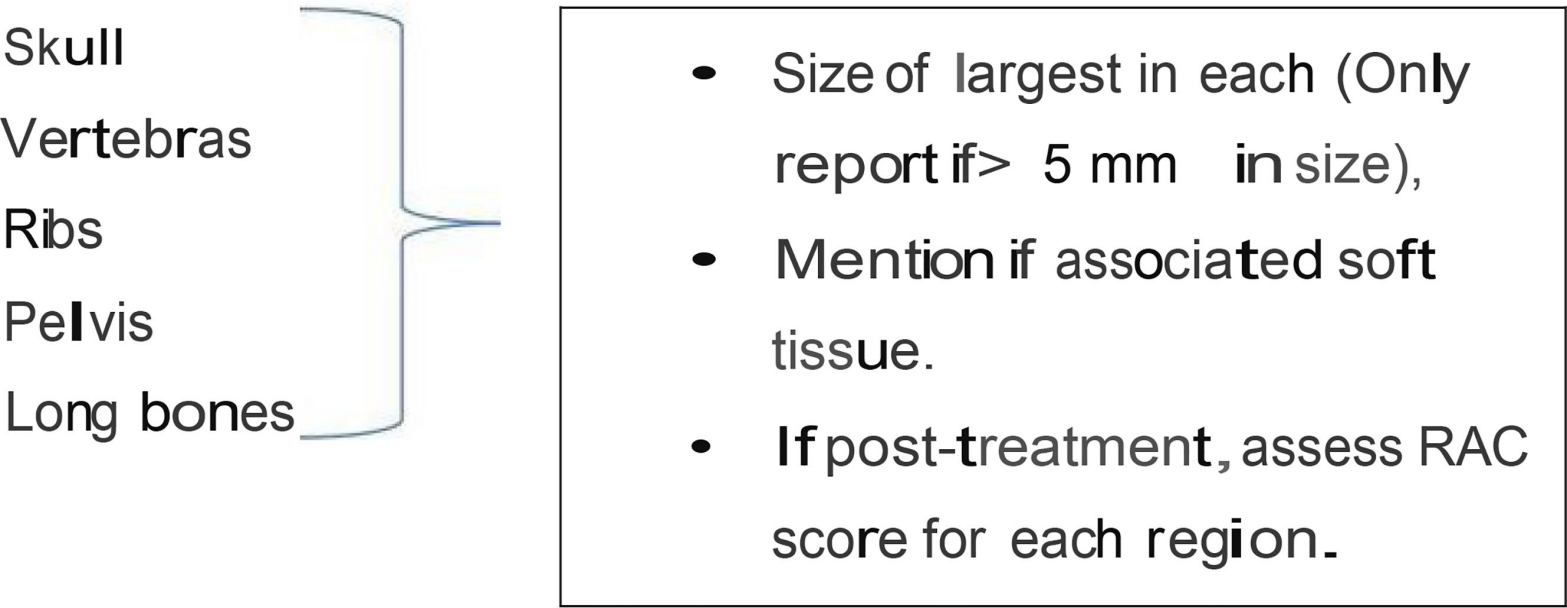	**Complications:** Fractures - Further cha racterise Benign/Malignant; AVN/ONJ. Cord/Nerve root compression, marrow reconversion.
	**Posterior Iliac crests:** Whether trephine likely to be representative
	**Other Findings:**
	Brain
	Neck
	Lungs
	**Abdominal organs:** Liver, Spleen, B/L Kidneys, Peri toneum, Pancreas
Pattern of predominant narrow infiltration (Normal/focal, focal on diffuse, salt pepper]	**Conclusions:** Summarise findings, MY-RADS score (if response assessment

**Table 6:** MY-RADS response assessment categories.

1	Response; highly likely	Unequivocal decrease in size and number of lesions/soft tissue component Return of fat in vertebra or within/around lesions ADC >1,400 µm^2^/s ≥40% increase in ADC from baseline with decrease in *b*-value
2	Response; likely	Small decrease in size/number of focal lesions Increase in ADC from ≤1,000 to <1,400 µm^2^/s >25% but <40% increase in ADC from baseline
3	Stable disease	No obvious change
4	Progression; likely	Equivocal new lesions Decreasing ADC Re-emergence of previously disappeared lesions
5	Progression; highly likely	New-onset pathological critical fracture(s)/cord compression requiring radiation therapy/surgery Unequivocal new focal/diffuse infiltration Unequivocal increase in number/size of focal lesions Evolution of focal lesions to diffuse neoplastic pattern Appearance/increasing soft tissue associated with bone disease New lesions/regions of high *b*-value with ADC values between 600 and 1,000 µm^2^/s

For prognostication, >7 lesions worse prognosis.

## Radiological Mimics and Differential Diagnosis

The list of lesions that can look like MM on imaging is extensive and depends on the type of presentation of myeloma, whether solitary, focal, or diffuse; the differential varies as enumerated in **Table 7**. Imaging alone may not suffice to make an accurate diagnosis between metastasis and myeloma and they remain close differentials (25). In the correct clinical-biochemical setting, we may favor the diagnosis of one over the other. Similarly, an appropriate clinical and laboratory context is needed to differentiate a few of the other entities described in the table above. For example, lymphoma spine sclerosis post-treatment can look like POEMS on CT which also causes diffuse bony sclerosis; however, their hematological and bone marrow findings are so different that a clinician would be seldom confused between the two (30).

**Table 7:** Differential diagnosis for imaging features of various plasma cell dyscrasias.

Differentials to consider for multifocal marrow Lesions	Differentials to consider for skull lesions	Differentials to consider for a solitary plasmacytoma
*Metastases*—prime differential Posterior vertebral element involvement (e.g., pedicles) favors metastases. Uncommon with myeloma due to paucity of red marrow Non-uniformity of lesions favors metastases Mandibular involvement—favors myeloma Presence of “mini-brain” sign—favors myeloma (thick bony struts radiating inwards)	*Calvarial metastases* Metastases usually are of varying sizes, with poor zone of transition Myeloma lesions are sharp punched out defects that tend to be more uniform in size Endosteal scalloping classic for myeloma	Expansile metastases “*blowout metastases*” For example in thyroid cancer, renal cell carcinoma, and choriocarcinoma
Other *marrow replacing lesions*, such as: Lymphoma Amyloidosis Waldenstrom macroglobulinemia—may have associated bone infarcts (due to the hyperviscosity)	*Foveolae/prominent arachnoid granulations* Involve inner table and diploae, sometimes reaching up to the outer cortex CSF density/intensity Smoothly marginated	Other causes of “soap-bubble” appearance Giant cell tumor Brown’s tumor—when history and other features of secondary hyperparathyroidism are present in the setting of CKD
*Osteoporotic fractures* Exclusively affects the vertebral body without any associated soft tissue Concave posterior wall, with altered marrow signal restricted to vertebral body	*Systemic disorders* Histiocytosis, for example LCH–calvarial lesions showing “bevelled” edges and other features such as pulmonary cysts Renal osteodystrophy/hyperparathyroidism—salt-and-pepper skull	

## Other Plasma Cell Dyscrasias

### Plasmacytoma

Plasmacytoma is characterized by a focal plasma cell proliferation in the absence of disease manifestations elsewhere. It may arise from osseous tissue as “solitary bone plasmacytoma” or soft tissues of the body as “solitary extramedullary plasmacytoma”. Solitary bone plasmacytomas are more common (70%) and bear a poorer prognosis than the extramedullary subtype (20), with a higher risk of progression to multiple myeloma (35%) compared with extramedullary lesions (7%) (87).

**Figure 8:**
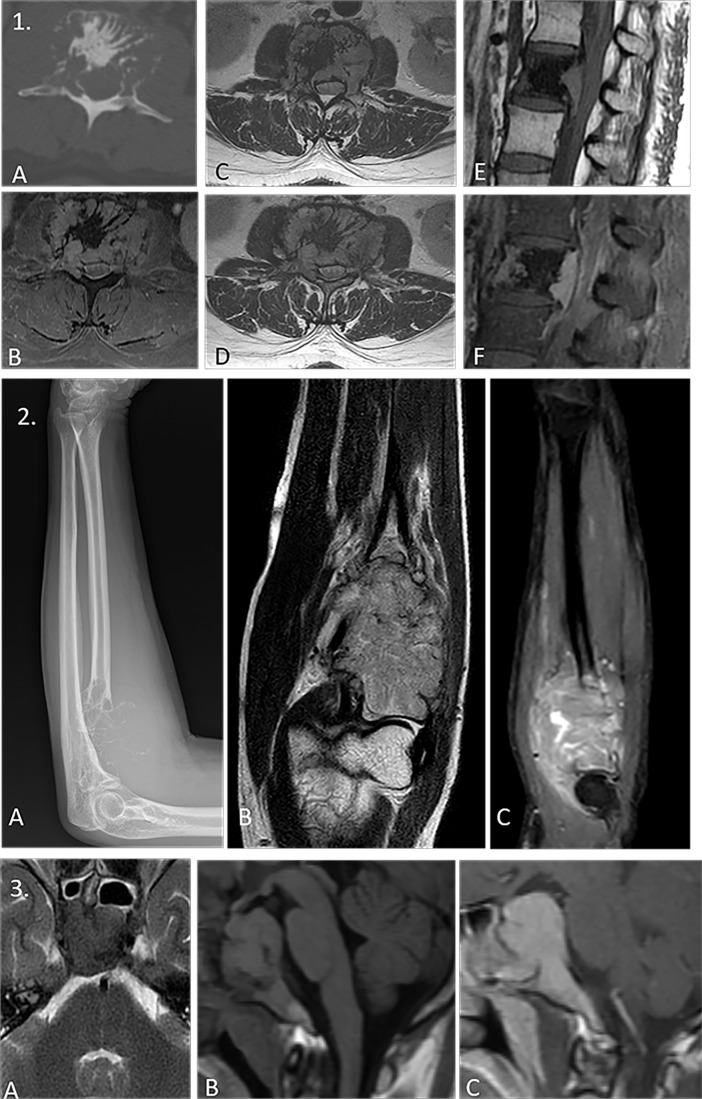
(1) Panel of images demonstrating the classic “mini-brain” appearance in a case of plasmacytoma on therapy with multiple hypointense struts seen emanating from the L2 vertebral body lesion seen on axial computed tomography (CT) [image **(A)**], axial STIR [image **(B)**], axial T1WI [image **(C)**], and axial T2WI [image **(D)**]. Note the central sclerosis representing post-therapy changes. The non-sclerotic component shows intense post-contrast enhancement with retropulsion as seen in pre-contrast sagittal T1W [image **(E)**] and post-contrast sagittal images [image **(F)**]. (2) Solitary plasmacytoma mimicking a soap-bubble lesion. A large expansile multilobulated lesion is seen involving the proximal radius on the radiograph [image **(A)**]. Coronal T2WI MRI image shows an intermediate T2 signal intensity lesion [image **(B)**] which shows post-contrast enhancement [image **(C)**]. Differentials of such a radiologic appearance include the giant cell tumor and blowout metastases. (3) Expansile lesion involving the clivus with T2 iso- to hypointense signal intensity [image **(A)**], T1 isointense signal intensity [image **B**], and intense post-contrast enhancement [image **(C)**]. The lack of T2 hyperintensity makes both chordoma and chondrosarcoma (lesions classically affecting the clivus) highly unlikely. Endonasal sampling revealed features of plasma cell neoplasm.

It is imperative to exclude additional osteolytic or soft tissue lesions so as to preclude the diagnosis of frank multiple myeloma (88, 89) (**Figure 8**).

PET/CT remains the modality of choice for staging workup of extramedullary plasmacytoma, while WBMRI is preferred for solitary bone lesions.

Solitary bone plasmacytomas appear as expansile, lytic lesions with marked cortical thinning and expansion. Occasionally, the “mini-brain” appearance may be noted where curvilinear low signal intensity struts may be seen radiating from the lesion (90, 91). In advanced stages, it may appear as a “soap-bubble” lesion, with a similar appearance to a giant cell tumor or Brown’s tumor.

### POEMS

POEMS is a multisystemic syndrome associated with plasma cell dyscrasia that consists of polyneuropathy, organomegaly, endocrinopathy, monoclonal gammopathy, and skin changes (92). It is responsible for less than 1% of myeloma cases. It shows a 5-year survival of 60% and has a better prognosis than that of multiple bone myeloma. Osteosclerotic lesions are present in approximately 95% of patients and are easy to confuse with enostosis or fibro-osseous bone lesions. The lesions may be densely sclerotic or have a sclerotic rim around a lucent center (unlike the typical myeloma lesions). CT is better for the overall evaluation of these lesions as they only inconsistently demonstrate FDG avidity (93) (**Supplementary Figure 2**).

### Waldenstrom Macroglobulinemia

It is a chronic, indolent monoclonal gammopathy with multiple features including anemia, high blood viscosity, lymphadenopathy, increased serum globulins, and bone marrow infiltration by lymphocytoid cells (94). MRI being the gold standard for marrow involvement is the most useful for the detection of bony changes, which may be diffuse or variegated. Focal lesions are rare. The presence of bone infarcts due to underlying hyperviscosity is classic, when present (95).

## Conclusions

Imaging plays a pivotal role in the diagnosis and management of plasma cell-related bone disease. The NICE and British guidelines suggest WBMRI as the initial screening modality, while IMWG recommends WBMRI in only those patients in whom WBLDCT screening is negative, due to the costs and technical intricacies involved in WBMRI. 18F-FDG PET/CT is the ideal response assessment tool, though further clinical trials comparing functional MRI versus PET/CT are the need of the hour. Newer advances like PET/MRI and novel radiotracers are also developments to watch out for in this field that may revolutionize future management.

## Author Contributions

All authors listed have made a substantial, direct, and intellectual contribution to the work and approved it for publication.

## Funding

The APCs are funded by an intramural sanction from the academic funds of Tata Memorial Centre, Mumbai, India.

## Conflict of Interest

The authors declare that the research was conducted in the absence of any commercial or financial relationships that could be construed as a potential conflict of interest.

## Publisher’s Note

All claims expressed in this article are solely those of the authors and do not necessarily represent those of their affiliated organizations, or those of the publisher, the editors and the reviewers. Any product that may be evaluated in this article, or claim that may be made by its manufacturer, is not guaranteed or endorsed by the publisher.

## Supplementary Material

Supplementary Figure 1 Differences between benign and malignant vertebral fractures on imaging.

Supplementary Figure 2 (1) Imaging mimics of myeloma skull lesions. Images **A–C** show few well defined excavations in the inner skull table with regular, well corticated margins and no soft tissue associated with them. These are prominent intraosseous arachnoid granulations or foveolae, and must not be confused for lytic lesions. These typically occur in vicinity of the venous sinuses and on MRI these show CSF signal intensity. Images **D–G, D** shows a well-defined intraosseous lytic lesion which shows a mean density of about -26 HU (Image). On MRI, the lesion is hyperintense on T1WI with suppression of the fat on T1+c Fat saturated images, representing an intraosseous lipoma. It is imperative to measure density routinely so as not to overcall myeloma lesions. (2) Panel of images demonstrating the skeletal findings in POEMS seen as diffusely sclerotic calvarial thickening (Image **A**), with few punctate osteosclerotic foci (arrows in **B**). The sclerotic lesions in POEMS may not show FDG avidity on PET/CT scans unless associated with a lytic/soft tissue component, as demonstrated in images **C, D** wherein the posteriorly located non-sclerotic part of the lesion shows FDG uptake. Thus, careful scrutiny of the CT component of PET/CT is important in identifying sclerotic POEMS lesions.

## Abbreviations

MM, multiple myeloma; SMM, smoldering multiple myeloma; MRI, magnetic resonance imaging.
