# Supplementary figures and images for: Imaging for Plasma Cell Dyscrasias: What, When, and How?
Source: Front Oncol. 2022 Mar 24;12:825394. doi: 10.3389/fonc.2022.825394 (PMC8987930; doi:10.3389/fonc.2022.825394)

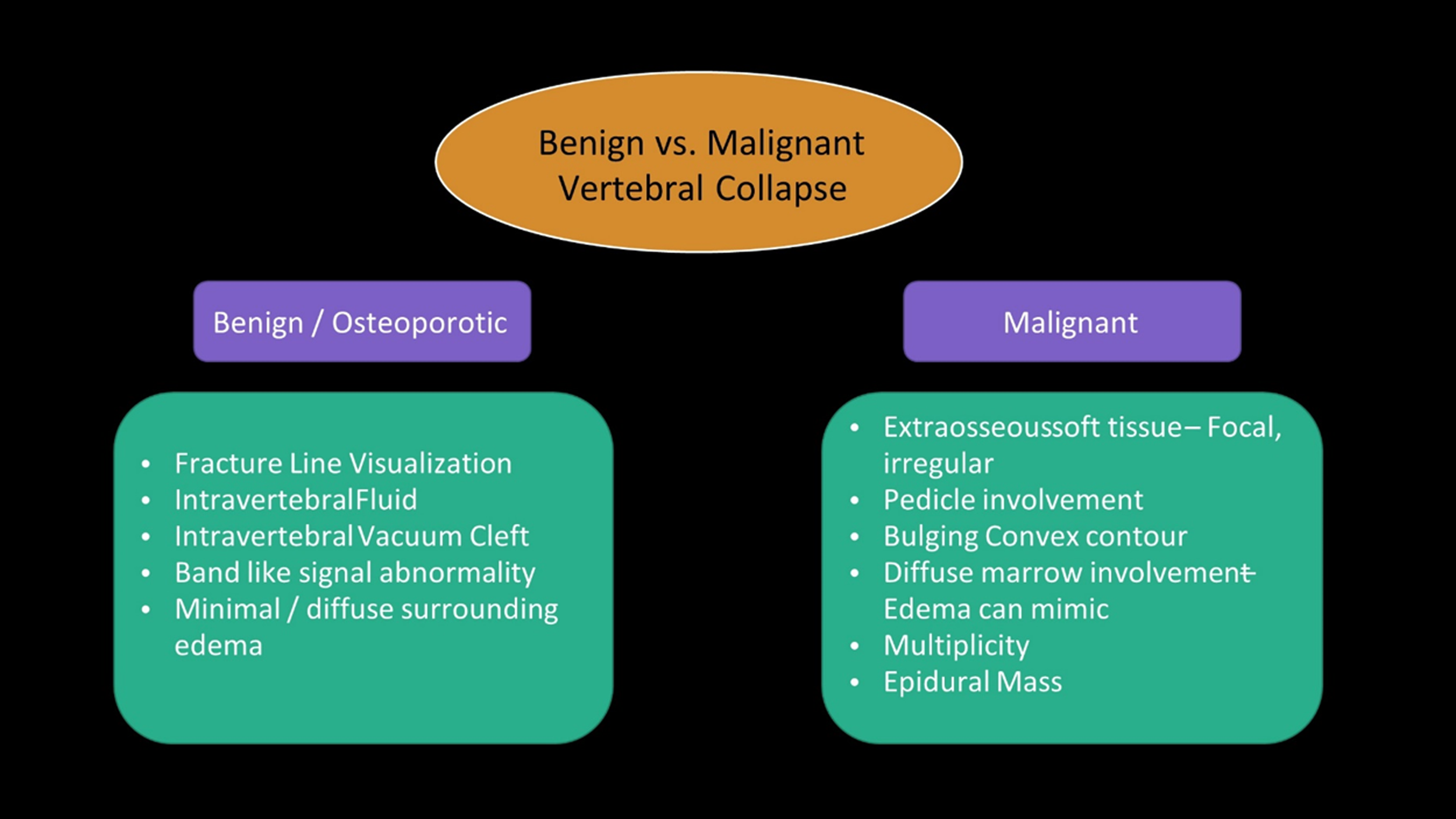

Supplement: Supplementary Figure 1 — Differences between benign and malignant vertebral fractures on imaging. [file Image_1.tif]

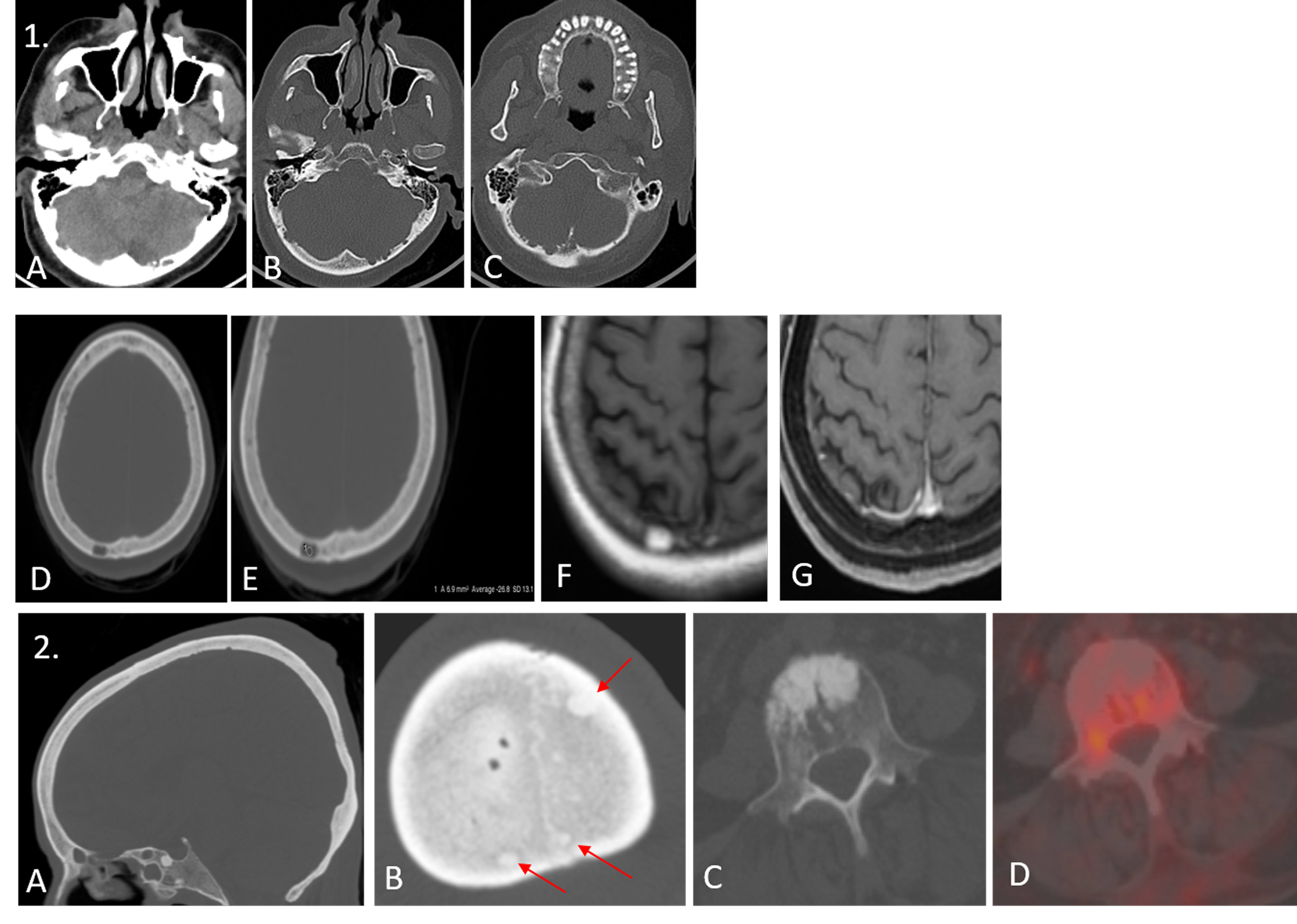

Supplement: Supplementary Figure 2 — (1) Imaging mimics of myeloma skull lesions. Images A–C show few well defined excavations in the inner skull table with regular, well corticated margins and no soft tissue associated with them. These are prominent intraosseous arachnoid granulations or foveolae, and must not be confused for lytic lesions. These typically occur in vicinity of the venous sinuses and on MRI these show CSF signal intensity. Images D–G, D shows a well-defined intraosseous lytic lesion which shows a mean density of about -26 HU (Image). On MRI, the lesion is hyperintense on T1WI with suppression of the fat on T1+c Fat saturated images, representing an intraosseous lipoma. It is imperative to measure density routinely so as not to overcall myeloma lesions. (2) Panel of images demonstrating the skeletal findings in POEMS seen as diffusely sclerotic calvarial thickening (Image A), with few punctate osteosclerotic foci (arrows in B). The sclerotic lesions in POEMS may not show FDG avidity on PET/CT scans unless associated with a lytic/soft tissue component, as demonstrated in images C, D wherein the posteriorly located non-sclerotic part of the lesion shows FDG uptake. Thus, careful scrutiny of the CT component of PET/CT is important in identifying sclerotic POEMS lesions. [file Image_2.tif]
